# A role for the fusogen *eff-1* in epidermal stem cell number robustness in *Caenorhabditis elegans*

**DOI:** 10.1038/s41598-021-88500-4

**Published:** 2021-05-07

**Authors:** Sneha L. Koneru, Fu Xiang Quah, Ritobrata Ghose, Mark Hintze, Nicola Gritti, Jeroen Sebastiaan van Zon, Michalis Barkoulas

**Affiliations:** 1 Department of Life Sciences, Imperial College, London, SW7 2AZ UK; 2 AMOLF, Science Park 104, 1098 XG Amsterdam, the Netherlands; 3 Present Address: Centre for Genomic Regulation (CRG), The Barcelona Institute of Science and Technology, Dr. Aiguader 88, 08003 Barcelona, Spain

**Keywords:** Caenorhabditis elegans, Differentiation, Development

## Abstract

Developmental patterning in *Caenorhabditis elegans* is known to proceed in a highly stereotypical manner, which raises the question of how developmental robustness is achieved despite the inevitable stochastic noise. We focus here on a population of epidermal cells, the seam cells, which show stem cell-like behaviour and divide symmetrically and asymmetrically over post-embryonic development to generate epidermal and neuronal tissues. We have conducted a mutagenesis screen to identify mutants that introduce phenotypic variability in the normally invariant seam cell population. We report here that a null mutation in the fusogen *eff-1* increases seam cell number variability. Using time-lapse microscopy and single molecule fluorescence hybridisation, we find that seam cell division and differentiation patterns are mostly unperturbed in *eff-1* mutants, indicating that cell fusion is uncoupled from the cell differentiation programme. Nevertheless, seam cell losses due to the inappropriate differentiation of both daughter cells following division, as well as seam cell gains through symmetric divisions towards the seam cell fate were observed at low frequency. We show that these stochastic errors likely arise through accumulation of defects interrupting the continuity of the seam and changing seam cell shape, highlighting the role of tissue homeostasis in suppressing phenotypic variability during development.

## Introduction

Development of multicellular organisms requires coordination of cell division and differentiation events across spatial and temporal scales to produce functional organisms in changing environments. Development can be robust to internal and external perturbations, such as genetic mutations or changes in nutrition and temperature^1,2^. Some perturbations are inevitable, for example gene expression variability that is present even in isogenic cells growing in a well-controlled environment, making robustness an indispensable property of biological systems^3–5^. As a result, a key challenge in developmental biology is to understand the mechanistic basis of biological robustness to different perturbations including molecular stochasticity.

*C. elegans* is an excellent model to study developmental robustness at the cellular level. The entire embryonic and post-embryonic cell lineage is well known and is mostly invariant from animal to animal in the population^6,7^. *C. elegans* is easy to culture under constant laboratory conditions and the animals are isogenic, which minimises the confounding effect of background genetic variation and environmental variation in studying robustness. We focus here on seam cells, a population of epidermal cells that show stem cell properties^8^. The *C. elegans* L1 larvae are born with 10 seam cells per lateral side. These cells undergo stereotypical symmetric and asymmetric cell divisions during larval development. Symmetric division occurs once in the early L2 stage, when H1, V1–V4 and V6 seam cells divide and both daughter cells retain the seam cell fate. Asymmetric cell divisions are reiterative throughout development, and the most common case involves an anterior daughter cell that differentiates into a neuron or epidermal cell, while the posterior daughter retains the seam cell fate. These division and differentiation patterns give rise to 16 seam cells per lateral side in wild-type animals at the end of larval development, a number which is robust in standard growth conditions, although it can be sensitive to temperature increase^9,10^.

Cell-to-cell fusion is a fundamental process that shapes development in many animals including humans, where fusion plays a role for example in muscle fibre formation and fusion of epithelial cells in the placenta^11,12^. In *C. elegans*, approximately one-third of the somatic cells generated during development undergo fusion to form multinucleated syncytia^13^. The epidermis is a good example of this process because it is made of 8 syncytia containing 186 nuclei in total, with hyp7 being the largest syncytium surrounding the majority of the animal body and containing 139 nuclei^14^. Two genes, *eff-1* and its paralog *aff-1*, encode nematode-specific transmembrane proteins that share structural similarities with viral fusogens and are required for most cell fusion events in *C. elegans*^15–17^. In the seam, EFF-1 is essential for the fusion of the anterior seam cell daughters after asymmetric cell division to the hyp7 syncytium during larval development^13,18^, while AFF-1 is required for the fusion between seam cells at the L4 larval stage, which is associated with their terminal differentiation. Ectopic *eff-1* expression causes fusion between cells that are not normally fated to fuse^16^. Given how potent fusogens are, tight transcriptional regulation is of paramount importance to provide spatiotemporal control of fusion events during development. In seam cells, GATA transcription factors such as ELT-1 and EGL-18 or the *C. elegans* homolog of *engrailed* CEH-16 are thought to repress *eff-1* during embryonic and post-embryonic development to prevent inappropriate fusion^19–22^. During asymmetric seam cell divisions, EFF-1 becomes enriched at fusion sites soon after the anterior seam cell daughter is born^23^. In *eff-1* mutants, anterior seam cell daughters do not fuse to hyp7 and therefore fail to join the syncytium^15^. However, the developmental fate that these epidermal cells acquire when fusion fails is still not well understood^15,21^.

We investigate here how phenotypic variability emerges in the *C. elegans* epidermis and report that *eff-1* loss-of-function mutants display an increase in seam cell number variability. Long-term time-lapse lineaging and single molecule fluorescent in situ hybridisation suggest that the patterns of cell division and differentiation in the *eff-1* mutant epidermis are largely unperturbed. However, developmental patterning errors occur at low frequency and these contribute to changes in seam cell number, while they are associated with broader defects in seam cell shape and tissue continuity. Our study provides an example of a mutation in a core gene network component, which is able to influence phenotypic variability through non cell-autonomous effects on tissue homeostasis.

## Results

### Mutations in the fusogen *eff-1* lead to increase in seam cell number variability

**Figure 1:**
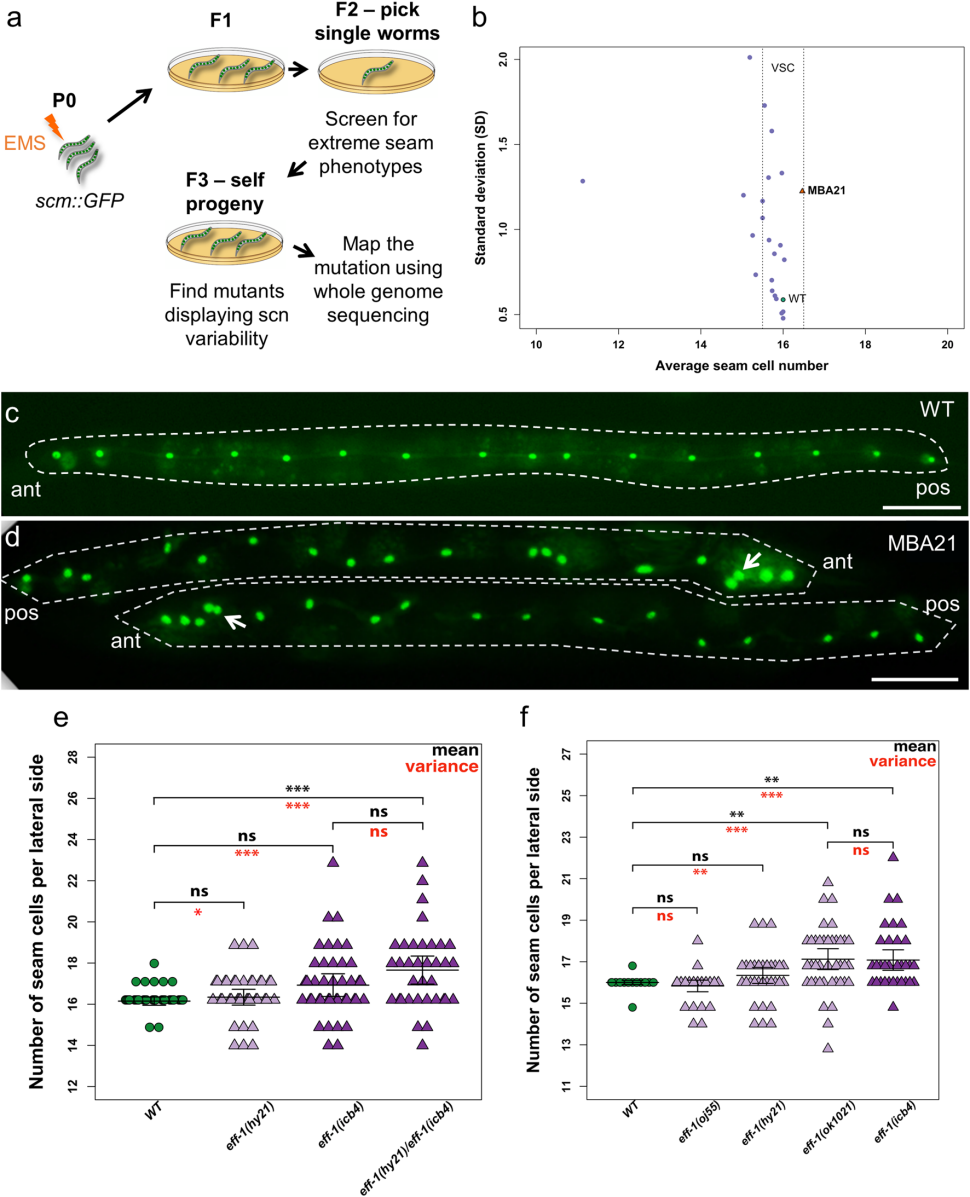
The *icb4* mutation represents a strong loss of function allele of *eff-1* and leads to seam cell number variability. (**a**) Design of the genetic screen to recover variable seam cell mutants. (**b**) Relationship between average seam cell number (SCN) and standard deviation (SD). Each point represents a mutant from our EMS screen. Parental wild-type strain (JR667) and mutant strain (MBA21) are shown in green and orange respectively, n ≥ 26 animals per strain. Mutants with similar average SCN but higher SD compared to wild type (indicated by the vertical dotted lines) were considered variable seam cell (VSC) mutants. (**c**–**d**) Representative images of wild-type (**c**) and MBA21 mutants (**d**) at the L4 stage. Note that MBA21 animals are dumpy compared to the wild-type and show an uneven distribution of seam cells towards the head. White arrows indicate clusters of seam cells within the head region. Scale bars are 50 µm and ant, pos stand for anterior and posterior side of the animal respectively. (**e**) *icb4* fails to complement the *hy21* mutant allele of *eff-1* (Missense P183L), 33 ≤ n ≤ 41. A one-way ANOVA showed that the effect of strain on SCN was significant (F (3, 143) = 7.23, *p* = 2 × 10^−4^). (**f**) Seam cell number variability is increased in severe *eff-1* loss-of-function mutants, 30 ≤ n ≤ 40. A one-way ANOVA showed that there was a significant effect of strain on seam cell number (F (4, 170) = 8.67, *p* = 2.17 × 10^–6^). In e and f, error bars indicate 95% confidence intervals. Black stars show statistically significant changes in the average seam cell number by post hoc Tukey’s HSD test, and red stars depict changes in variance with a Levene’s median test (*** corresponds to *p* value < 1 × 10^−4^, **p < 1 × 10^−3^, *p < 0.05). Panels c–d were created using Fiji 2.0.0^44^ and panels b,e,f using R version 4.0.3^45^.

We have previously described a genetic strategy to identify genes influencing developmental variance focusing on seam cell number (SCN) as the quantitative phenotype of interest^9^. Briefly, we mutagenized a strain carrying the seam cell marker *scm::GFP* (*wIs51*), which is commonly used to visualise seam cells. We then isolated F2 animals showing deviations from the wild-type seam cell number range, that is animals displaying seam cell counts greater than 17 or fewer than 15 cells per lateral side. F2 mutant animals were allowed to produce self-progeny and seam cell number was scored in the F3 generation (Fig. 1a). We were particularly interested in identifying mutants that showed an increase in seam cell number variance without a change in the mean, which would be indicative of developmental variability introduced within the isogenic population (Fig. 1b). One of the mutations that we recovered from this screen was the recessive *icb4* in strain MBA21, which showed a significant increase in seam cell number variance compared to wild type (wild-type SCN = 16 ± 0.26 S.D. versus MBA21 SCN = 16.47 ± 1.22 SD, *p *_*variance*_ = 0.01, *p *_*mean*_ = 0.75, Fig. 1b). Mutant animals frequently displayed clusters of seam cells in the head region, as observed in 22% (9 out of 41) of MBA21 animals compared to 0% in wild type (Fig. 1c,d, white arrows).

To identify the causative mutation underlying seam cell number variability in MBA21, we crossed this strain to the polymorphic isolate CB4856 from Hawaii. The progeny of F2 recombinants displaying the variable phenotype were pooled together and sequenced. Using the CloudMap analysis pipeline^24^, we found that the mutation in MBA21 mapped to a region in the middle of chromosome II (Fig. S1, blue arrowhead). This region contained a C to T transition in the third exon of *eff-1* that results in a premature stop codon (Q148STOP). This represented a strong candidate to investigate further because *eff-1* encodes a fusogen that is required for most cell fusion events that occur during *C. elegans* development^15^, including the fusion of cells in the context of the epidermis. Furthermore, MBA21 mutant animals were smaller in size compared to wild type (Fig. 1c,d), which is consistent with what has been previously reported for other *eff-1* loss-of-function mutants^15,25^.

To validate that the *icb4* allele is indeed a new allele of *eff-1*, we performed genetic complementation using the previously characterised *eff-1(hy21)* mutant allele^15^. F1 hermaphrodites carrying *icb4* and *hy21* in *trans* displayed the variable seam cell number phenotype (Fig. 1e). One-way analysis of variance (ANOVA) confirmed that there is a significant effect of strain on the seam cell number (F (3, 143) = 7.23, *p* = 2 × 10^−04^). Post hoc Tukey HSD tests showed that seam cell number in *eff-1(hy21/icb4)* animals is significantly different from wild type suggesting that *icb4* does not complement *hy21* (*p* < 0.0004). We also observed that there was a more pronounced effect on seam cell variance in *eff-1(icb4)* and *eff-1(hy21/icb4)* compared to the hypomorphic allele *eff-1(hy21)* (Fig. 1e). Taken together, these results indicate that *icb4* is a new, strong loss-of-function allele of *eff-1*. An increase in seam cell number variance in *eff-1(icb4)* animals compared to wild type was also observed at 25 °C (Fig. S2). However, seam cell number and variance in wild type is mildly increased at 25 °C compared to 20 °C as previously reported^10^, therefore we chose to use 20° as the temperature for all our experiments.

To understand how the severity of *eff-1* loss-of-function correlates with phenotypic variability, we quantified seam cell number in backgrounds carrying *eff-1* alleles of different strength. We found that animals carrying the strong loss-of-function allele *ok1021,* which is thought to be functionally null^17^, have a significant difference in seam cell number variability compared to wild type (*p* < 0.0001) and no difference (*p* = 0.97) in seam cell number or seam cell number variability in comparison to the *icb4* allele (Fig. 1f). Animals carrying the hypomorphic allele *hy21* show a milder increase in seam cell number variability compared to wild type, while animals carrying the weak loss-of-function allele *oj55* do not show a significant difference (*p* = 0.65) (Fig. 1f). These results indicate that seam cell variability becomes more pronounced as the severity of the *eff-1* mutation increases.

### Quantitative characterisation of seam cell patterning in *eff-1(icb4)* mutants

Cell fusion is fundamental for embryonic and post-embryonic development in *C. elegans*^15^. With regard to epidermal patterning, the reiterative post-embryonic asymmetric seam cell divisions usually produce anterior daughter cells that fuse to the main hypodermal syncytium (hyp7), while the posterior daughter cells maintain the seam cell fate. Loss of cell fusion can therefore disturb the syncytial nature of the epidermis. Interestingly, we found that *eff-1* loss-of-function individuals showed either a slight increase or decrease in the number of *scm::GFP* expressing cells compared to wild-type animals, which was not anticipated given the function of *eff-1* in driving fusion of differentiating cells that could potentially result in a large increase in seam cell number in the *eff-1* mutant background.

**Figure 2:**
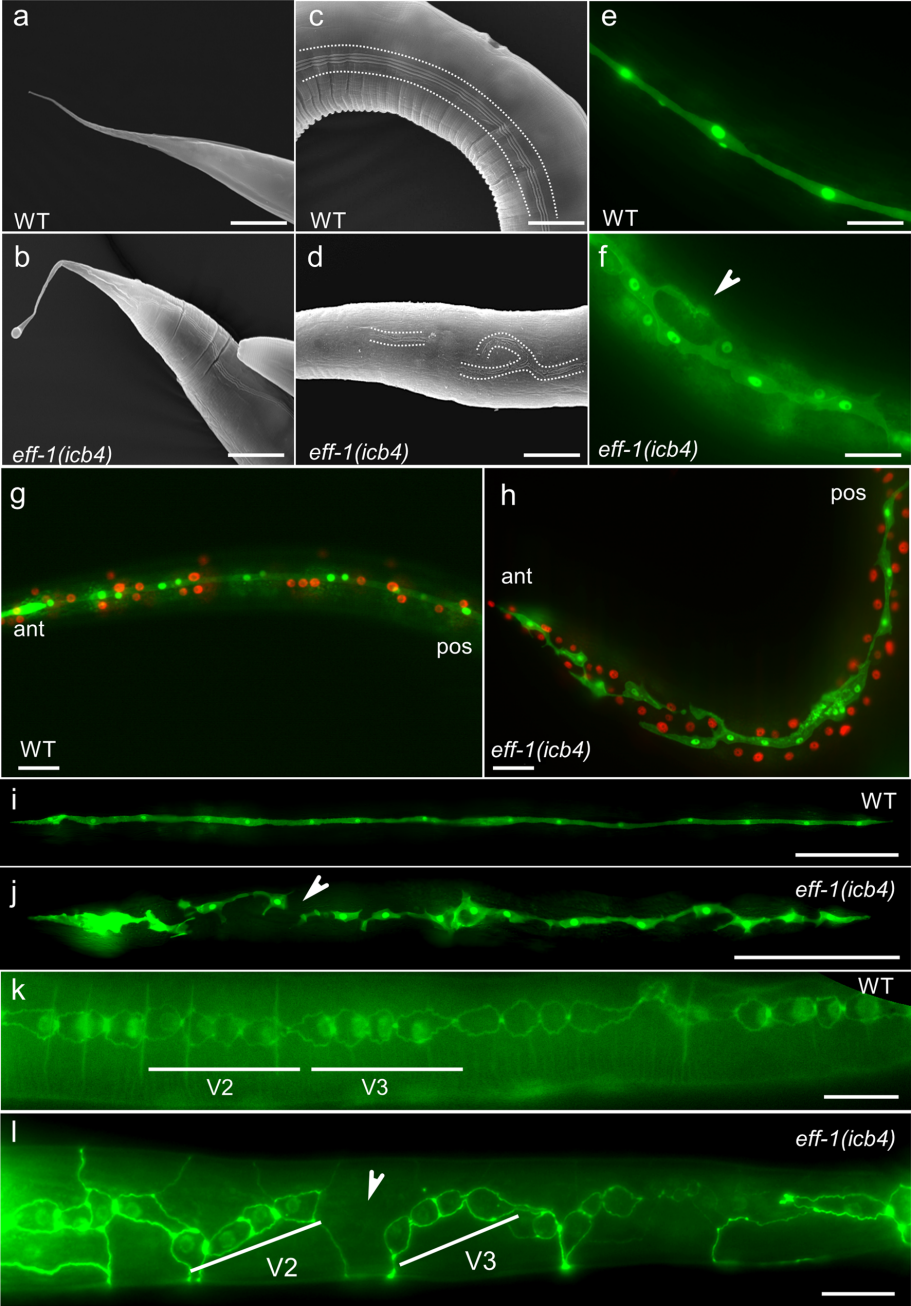
*eff-1(icb4)* animals display developmental defects in the tail, alae and seam. (**a**–**b**) Representative SEM images of tail spike in young adult wild-type and *eff-1(icb4)* animals respectively. Note that mutant animals have bulbous tail instead of smooth tail as in wild-type animals. (**c–d**) Representative SEM images of alae in young adult wild-type and *eff-1(icb4)* animals respectively. Mutant animals display cuticle defects and defective fragmented alae. (**e**–**f**) Seam cell defects in *eff-1(icb4)* late L4s in comparison to wild-type animals. Arrowhead points to a seam cell bifurcation in *eff-1(icb4)* (**f**), which does not occur in wild-type animals (**e**). (**g**–**h**) The expression of the *dpy-7p::mCherry* marker is not affected in the *eff-1* mutant background (**h**) in comparison to wild type (**g**). Ant, pos stand for anterior and posterior side of the animal, respectively. (**i**–**j**) Seam cells in *eff-1(icb4)* young adults are misaligned to each other and display gaps (arrowhead in j) in contrast to wild-type animals (**i**). (**k**–**l**) Loss of cell contact between V2 and V3 daughter cells in *eff-1(icb4)* at the L2 asymmetric cell division (**k**), marked with an arrowhead, in comparison to wild-type (**l**). Seam cells are visualised using the *scm::GFP* marker, as well as seam cell driven membrane-targeted GFP (GFP::CAAX)^10^) (**e**–**j**) or *ajm-1p::ajm-1::GFP* (**k**–**l**). Scale bars in a-h, k and l are 20 µm and in i-j 100 µm. All panels were created using Fiji 2.0.0^44^.

To investigate the phenotypic consequences of loss of *eff-1* function in the seam, we used fluorescence and scanning electron microscopy*. eff-1(icb4)* mutants displayed additional morphological abnormalities previously reported in other *eff-1* loss-of-function alleles^15^, such as a deformed tail spike. This phenotype was observed in 100% of *eff-1(icb4)* animals, which showed a bulbous tail compared to a tapered tail in wild type (Fig. 2a,b). Aberrant alae and seam cell distribution, often fragmented or bifurcated, were also observed in *eff-1(icb4)* mutants, unlike the linearly arranged and continuous wild-type seam cells and alae (Fig. 2c–f). Seam cells are dynamic and change their shape as they go through rounds of cell division. This is because the continuity of the seam line is interrupted post cell division due to cell differentiation of daughter cells, thereby requiring seam cells to change shape to re-establish a physical connection^26^. We reasoned that seam cell bifurcations in *eff-1(icb4)* animals may arise from cells that obstruct the re-establishment of connections since they stand in the middle, unable to fuse and move out of the seam line. To test this idea, we used a strain carrying the seam cell marker together with a hypodermal reporter where m*Cherry* is driven under the *dpy-7* promoter, and we observed that the continuity of the seam line was interrupted by the presence of *dpy-7* positive cells (Fig. 2g,h). Furthermore, we found frequent breaks in the continuity of the seam with 90% (36 out of 40) of *eff-1(icb4)* adults showing such breaks as opposed to 0% in wild-type animals (arrowheads in Fig. 2i–l). Interestingly, *eff-1* expression is confined to differentiating cells and the hypodermis, as opposed to cells that retain the seam cell fate (Fig. S3a-c), which suggests that loss of *eff-1* function may have broader phenotypic consequences at the tissue level.

**Figure 3:**
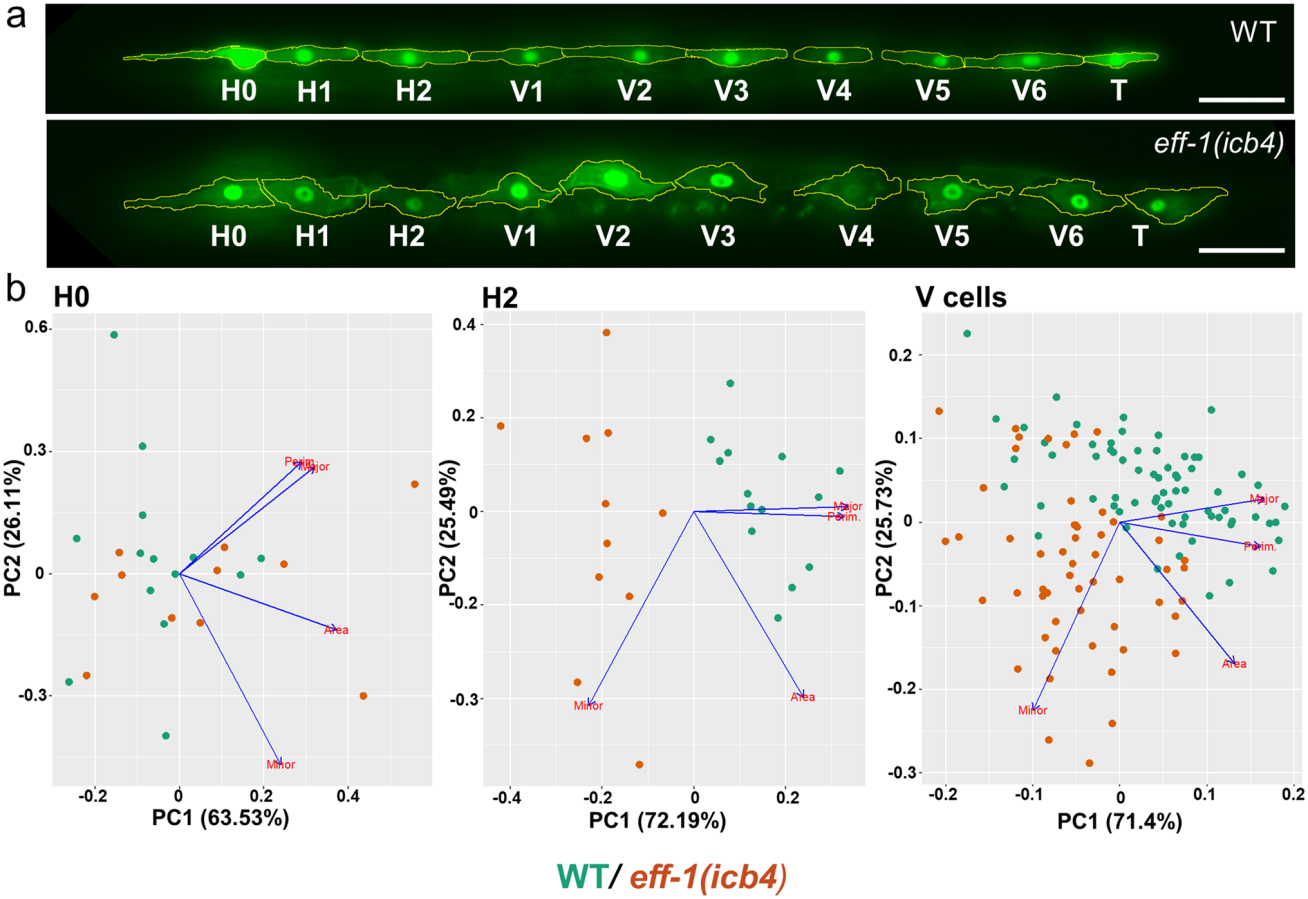
Seam cell shape defects in *eff-1(icb4)* mutants. (**a**) Representative images of seam cells in wild-type and *eff-1(icb4)* mutants after the L1 stage division, scale bar is 20 μm. Seam cells are visualised by membrane targeted GFP (GFP::CAAX) driven under the last intron of *arf-5* together with a minimal *pes-10* promoter and *scm::GFP.* (**b**) Comparison of seam cell shape between wild-type and *eff-1(icb4)* mutants. Green and orange dots correspond to seam cells in wild-type and *eff-1(icb4)* animals respectively. Individual cells are plotted with respect to first and second principal components, which account for more than > 90% of the total variance. Arrows represent variables in the PCA and point in the direction of increasing values of that variable (major/minor axis, perimeter, area). Note that H2 and V cells occupy distinct space in *eff-1(icb4)* is compared to wild type. Panel a was created using Fiji 2.0.0^44^ and panel b using R version 4.0.3^45^.

We then asked whether cellular compartmentalisation of the normally syncytial epidermis in the absence of *eff-1* dependent cell fusion has an impact on seam cell shape. To quantify cell shape in *eff-1(icb4)*, we measured descriptive cell shape parameters on individual or pooled seam cells (Fig. 3a,b and Fig. S4) upon completion of the asymmetric cell division at the L1 stage. These shape parameters included cell area, perimeter, minor and major cell axes and were used to perform principal component analysis (PCA). The first two components were sufficient to account for > 90% of the total variance of each cell. Cell shape for most seam cells, except for H0, was found to be affected in *eff-1(icb4)* animals compared to wild-type animals (Fig. 3b and Fig. S4). Seam cells in *eff-1(icb4)* were found to be less elongated along the anteroposterior axis, but more extended on the dorsoventral axis in comparison to wild type. Taken together, we conclude that seam cell shape is different in *eff-1(icb4)* mutants starting from early post-embryonic development and this may contribute to the developmental defects observed.

**Figure 4:**
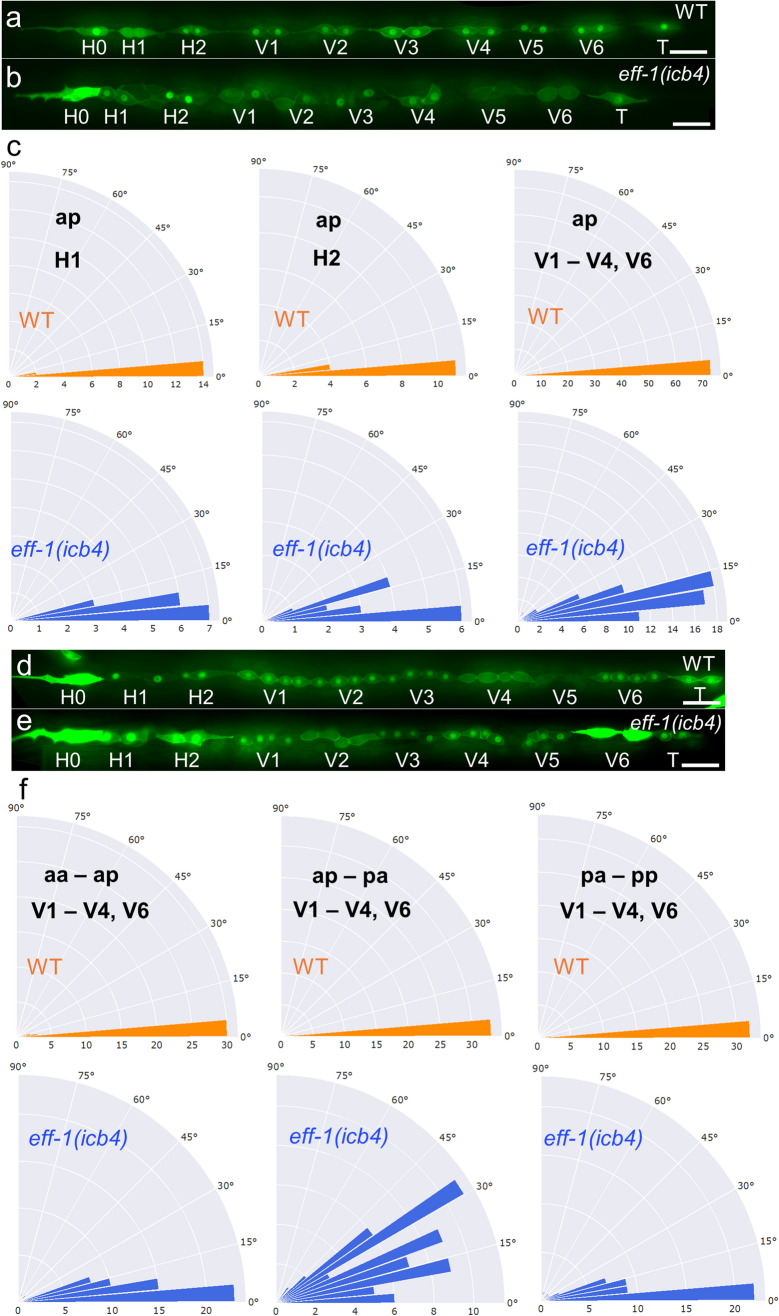
Seam cell angles are perturbed in *eff-1(icb4)* mutants. (**a–b**) Representative images of seam cells at the L2 symmetric division in wild-type (**a**) and *eff-1(icb4)* (**b**). Seam cells are visualised by membrane targeted GFP (GFP::CAAX) driven in seam cells and *scm::GFP.* (**c**) Rose plots showing angles between pairs of cells (**a**–**p**) in *eff-1(icb4)* and wild-type animals. A One-way ANOVA showed a significant difference (*p* < 0.006) in the angle between pairs of cells in *eff-1(icb4)* and wild-type (10 ≤ n ≤ 21 per cell for each strain). The angle between pairs of cells (**a** and **p**) is calculated as described in the materials and methods. (**d**–**e**) Representative images of wild-type and *eff-1(icb4)* seam cells at the L2 asymmetric cell division stage. (**f**) Angles of daughter cell position within pairs (aa / ap and pa / pp) and between adjacent pairs (ap-pa) of a lineage are calculated in a similar way. All rose plots show comparison between *eff-1(icb4)* and wild-type in frequency of phenotypic classes every 5º angle. A One-way ANOVA showed a significant difference (*p* < 0.001) in the angle between pairs of cells in *eff-1(icb4)*, 38 ≤ n ≤ 61 per cell pair and per strain. Scale bars in a, b, d and e are 20 µm. Panels a,b-d,e were created using Fiji 2.0.0^44^ and panels c,f using MATLAB 9.8 (https://uk.mathworks.com).

Elongated cell shape has been previously shown to be a key determinant of the axis of seam cell division^27^. Seam cells are arranged and divide in a linear manner in wild type with an angle between daughter cells (referred to as a-p for the symmetric L2 division) lesser than 11°. In contrast, we found a significant difference in the angle for H1, H2 and V(1-4,6) daughter cells between *eff-1(icb4)* and wild-type animals (Fig. 4a–c). V1–V4 and V6 undergo an additional asymmetric cell division at the late L2 stage giving rise to an anterior (simplified here as “aa/ap”) and posterior (“pa/pp”) cell pair. We measured the angle between daughter cells in *eff-1(icb4)* and wild-type animals within each pair (aa-ap and pa-pp) or between adjacent pairs (ap-pa) of the same lineage and also found a significant difference (Fig. 4d–f). Interestingly, there was a significant difference between the L2 symmetric (a-p) compared to the L2 asymmetric (ap-pa) angle in *eff-1(icb4)* mutants in contrast to no difference in wild-type (*p* = 3.26 × 10^−08^ in *eff-1(icb4)* vs *p* = 0.66 in wild-type). These results highlight that there is an increase in the misalignment of seam cells in the *eff-1* mutant background as development progresses.

Misalignment of cells in the *eff-1* mutant epidermis can result from a change in the angle of cell division or it may occur post cell division if cells are pushed out of alignment by the remaining unfused cells. To distinguish between these two possibilities, we measured the angle of cell division between segregating DNA in the anaphase of dividing seam cells at the L2 stage and the long axis of the cells, visualised by a GFP::H2B and membrane GFP::PH marker respectively both driven under a seam cell promoter^27^. We found no difference in cell division angles between the wild type and *eff-1(icb4)* animals (Fig. S5a–d). Therefore, we suggest that the misalignment observed in *eff-1* mutants is likely due to displacement of newly divided seam cells by unfused seam cells from previous cell divisions.

### Developmental basis of seam cell number variability in *eff-1(icb4)* mutants

**Figure 5:**
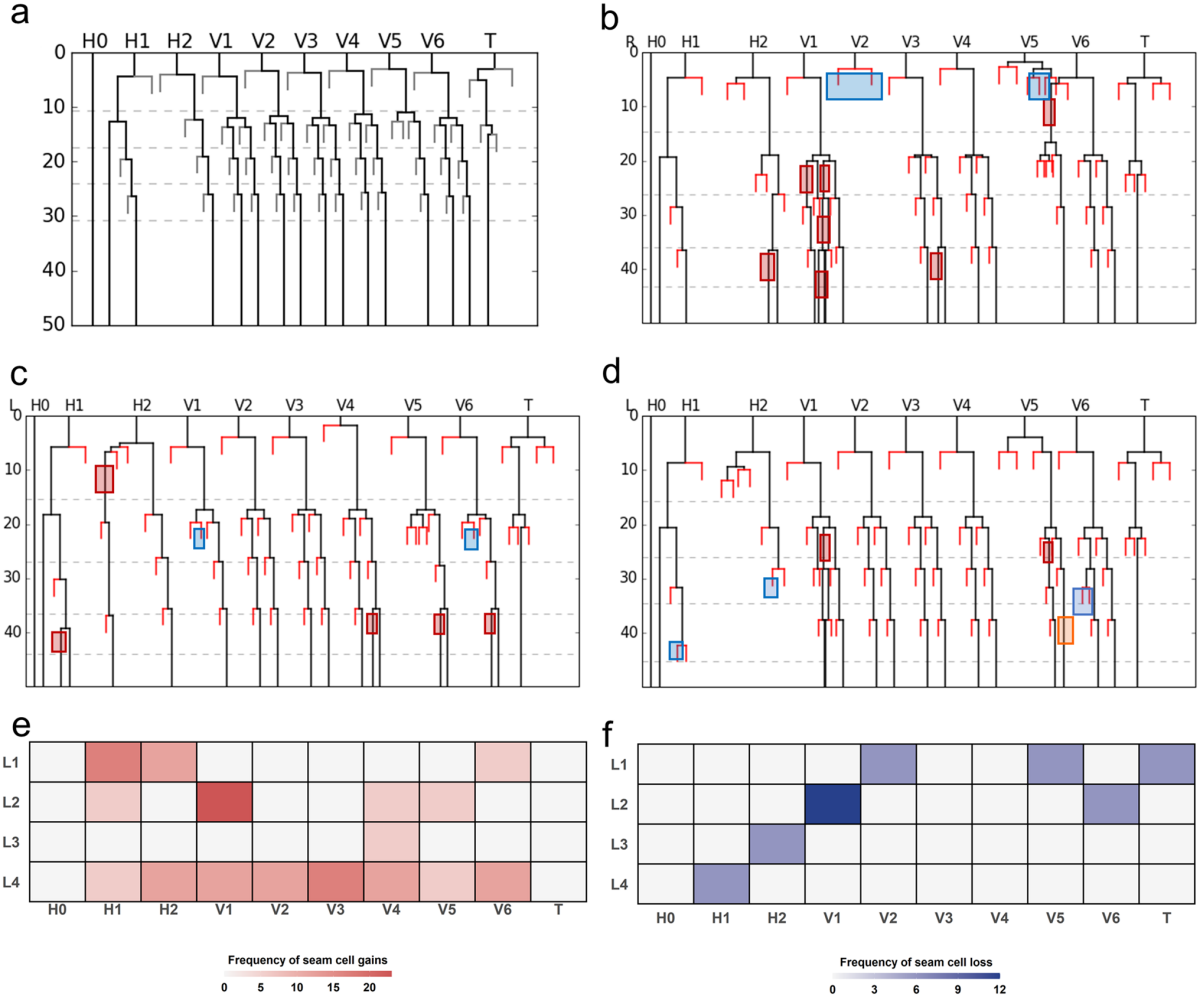
Developmental basis of seam cell number variability in *eff-1(icb4)*. (**a–d**) Representative seam cell lineages of wild-type (**a**) and *eff-1(icb4)* animals (**b**–**d**). The coloured boxes in (**b–d**) highlight developmental errors. Red boxes mark gains of seam cells, blue boxes loss of seam cells. Note that the animals in (**b–d**) show a terminal SCN of 19, 18 and 16 seam cells respectively. (**e**) Summary heat map of lineage errors observed which increase seam cell number. (**f**) Summary heat map of lineage errors which decrease seam cell number. Numbers in e and f represent percentage of the error in the 18 lineages analysed. Note that the percentage of symmetrisation errors towards the seam cell fate is highest at the L4 stage. Panels a-d were created using a lineaging visualisation pipeline described in^28^ and panels e–f using R version 4.0.3^45^.

To understand the developmental basis of seam cell number variability in *eff-1(icb4)* mutants, we performed long-term time-lapse imaging of postembryonic cell divisions in animals grown in microchambers from embryo to the early adult stage^28^. Wild-type and mutant animals hatched with 10 seam cells per lateral side (Fig. 5a–d). Despite the pronounced cell misalignment defects observed in the *eff-1(icb4)* background*,* we found that 39% (7 out of 18) seam cell lineages in the mutant maintained a wild-type seam cell number and overall division pattern. Nevertheless, we also observed patterning errors that change the seam cell number. First, we found frequent symmetrisation of cell division especially at the L4 stage towards the seam cell fate, which contributes to an increase in seam cell number (Fig. 5b–e). Consistent with the seam cell clustering phenotype in the head region of *eff-1(icb4)* animals, we found frequent symmetrisation of normally asymmetric cell divisions of H1 and H2 cells at the L1 stage (Fig. 5c,e). At the same time, we found rare seam cell losses at L1, L2 and L3 stages due to aberrant symmetric divisions producing two hypodermal cells (Fig. 5b–d,f). These errors were occasionally followed by symmetrisation favouring the seam cell fate in adjacent lineages at a later developmental stage (3/7 lineages, 43%), which compensated for the initial decrease in seam cell number. Taken together, our lineaging analysis reveals a low frequency of seam cell patterning errors upon loss of *eff-1.* These errors occur at all developmental stages and in all dividing lineages and have the potential to increase or decrease seam cell number depending on their type.

### Fusion is not required for differentiation of anterior seam daughters

**Figure 6:**
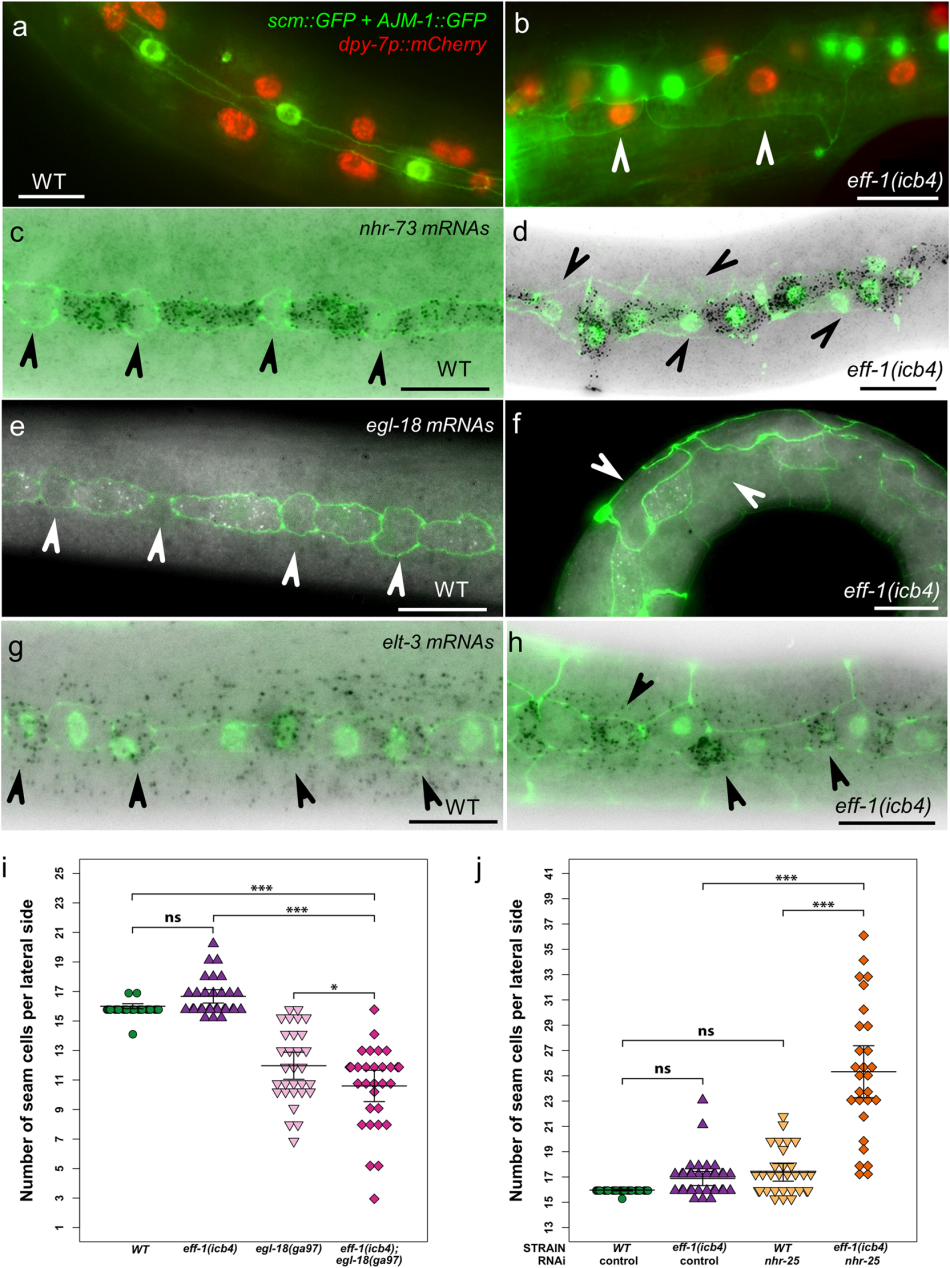
Anterior seam cell daughters still differentiate in the absence of fusion. (**a–b**) Representative images of wild-type and *eff-1(icb4)* animals expressing seam cell and hypodermal markers. *eff-1(icb4)* animals have epidermal cells showing ectopic *AJM-1::GFP* expression due to lack of fusion, while these cells show *dpy-7* marker expression (marked by arrowheads). (**c–h**) Representative smFISH images of *nhr-73* (**c**–**d**), *egl-18* (**e**–**f**) and *elt-3* (**g**–**h**) in wild-type and *eff-1(icb4)* animals. Note that posterior cells express seam cell-specific markers (*nhr-73, egl-18*) in *eff-1* mutants and anterior cells express the hypodermal marker *elt-3*. Seam cells are labelled in green using *ajm-1p::ajm-1::GFP* and *scm::GFP* and arrowheads point to differentiating cells. (**i**) Seam cell number increase in *eff-1* depends on EGL-18. One-way ANOVA shows there is a significant effect of strain on SCN (F (3, 116) = 67.4, *p* = 2.2 × 10^–16^). Post hoc Tukey HSD tests showed that there is a significant difference in SCN between *eff-1(icb4)* and double mutant *eff-1(icb4); egl-18(ga97) (p* < 1 × 10^−4^*,* n = 30 animals per strain). (**j**) Seam cell counts in the *eff-1(icb4)* mutant upon *nhr-25* RNAi reveal synthetic interaction between *eff-1* and *nhr-25*. One-way ANOVA shows there is a significant effect of strain on SCN (F (3, 117) = 67.01, *p* = 2.2 × 10^–16^). Post hoc Tukey HSD tests showed that there is a significant difference in SCN between *nhr-25* knockdown in *eff-1(icb4)* animals and control RNAi in *eff-1(icb4)* animals or *nhr-25* knockdown in wild-type (*p* < 1 × 10^−4^, 28 ≤ n ≤ 33 per strain). Error bars in i and j indicate 95% confidence intervals. Scale bars in a-h are 10 µm. Panels a-h were created using Fiji 2.0.0^44^ and panels i-j using R version 4.0.3^45^.

*eff-1* expression occurs in bursts in the differentiating daughter cells and is excluded from cells that maintain the seam cell fate (Fig. S3a)^9^. However, it remains unclear whether fusion is required for cell differentiation or whether cell differentiation is occurring independently of the differentiation programme. To resolve the developmental state of cells that do not fuse in *eff-1(icb4)* mutants, we used two approaches. First, we explored whether anterior daughter cells express hypodermal markers following seam cell division. We found that anterior daughters in *eff-1(icb4)* animals express *dpy-7p::mCherry* similar to wild-type animals, although they show expression of the apical junction marker *AJM-1::GFP* since they remain unfused and retain their borders (Fig. 6a,b, white arrowheads). Second, we used single molecule FISH to localise genes enriched in seam cells, such as the nuclear hormone receptor *nhr-73* and *egl-18*, as well as genes enriched in the hypodermis, such as the GATA transcription factor *elt-3*. We found *nhr-73* and *egl-18* expression in seam cell-fated cells in wild-type and *eff-1(icb4)* animals, as opposed to differentiating cells, suggesting that the asymmetric cell fate distribution is largely not perturbed (Fig. 6c–f, arrowheads point to differentiating cells). In addition, anterior daughters in *eff-1* mutants expressed the *elt-3* hypodermal marker similar to wild-type (Fig. 6g–h, arrowheads). Taken together, we conclude that anterior seam cell daughters are still largely able to differentiate in *eff-1(icb4)* animals despite their inability to fuse.

Anterior daughter cells, however, do occasionally acquire the seam cell fate at the expense of the hypodermal fate as revealed by the observed symmetrisation events in our lineage analysis. To address whether these symmetrisation events may depend on Wnt signalling, we studied the interaction with the POP-1/TCF downstream target EGL-18^22^. We found that double *eff-1(icb4)*; *egl-18(ga97)* mutants show a significant decrease in seam cell number in comparison to wild-type and *eff-1* mutants (Fig. 6i *p* < 0.01), indicating that maintenance and ectopic activation of seam cell fate in *eff-1(icb4)* requires Wnt pathway activity. Furthermore, we found a synthetic interaction with *nhr-25*, which encodes a nuclear hormone receptor known to regulate seam cell patterning and promote hypodermal differentiation^29–31^. We found a striking increase in seam cell number in *eff-1(icb4)* animals upon *nhr-25* knockdown in comparison to *eff-1(icb4)* animals on control RNAi bacteria or wild-type animals on *nhr-25* RNAi (Fig. 6j, *p* < *0.01*). This suggests that hypodermal cell differentiation in the *eff-1* mutant background is likely to depend on NHR-25 activity.

## Discussion

A key question in developmental biology is what makes biological systems robust to various types of perturbations^2^. Previous experimental evidence, mostly derived from studies in unicellular systems, has suggested that highly connected genes (also known as network hubs) are important modulators of phenotypic variance^32,33^. These highly connected components include molecular chaperones, such as Hsp90, which has been discussed in the context of buffering phenotypic variation in both animals and plants^34,35^. However, whether these factors will act as buffers or potentiators of phenotypic variability also depends on their interactions in gene regulatory networks^36^. Other commonly discussed contributors to phenotypic robustness are miRNAs^36^. Unbiased screens focusing on developmental variability as the trait of interest are more challenging to perform in plants and animals^9,37^, therefore the full spectrum of variance-influencing loci in multicellular eukaryotes and how these are integrated within developmental gene regulatory networks remain largely unknown^38^. Using a forward genetic approach, we studied here how seam cell number variability can emerge in an isogenic population. We demonstrate that a putative null mutation in the fusogen *eff-1* leads to a breakdown of developmental robustness in the *C. elegans* epidermis because it increases seam cell number variance. It is of note that the magnitude of the effect on variance, as well as the effect on the phenotypic mean, were found to depend on the strength of the mutant allele. This highlights that the definition of variance-influencing loci is also allele-specific, as it has been previously reported in other model systems as well^39^.

Based on our previous knowledge of epidermal development, it was unexpected to find seam cell number variability in *eff-1* loss-of-function mutants. This gene encodes a well-studied fusogen in *C. elegans* that plays a role in epidermal post-embryonic development by triggering the fusion of anterior seam cell daughters to the syncytial hypodermis following an asymmetric division^13–16^. We report here that developmental variability in *eff-1* mutants emerges through seam cell patterning defects, such as gains and losses of seam cells. Gains involve anterior daughters maintaining the seam cell fate post division, instead of differentiating into hypodermis, and this error is most frequently occurring in late larval stages. Losses of seam cells were attributed to hypodermal differentiation of both seam cell daughters following division. Given that *eff-1* expression is confined to differentiating cells and is excluded from seam cells, the effect on phenotypic variability must be exerted in a non-cell autonomous manner. Consistent with this idea, we found morphological changes in seam cell shape, with seam cells being misshapen and less elongated in the *eff-1* mutant background compared to the wild type, which is indicative of broader developmental defects at the tissue level.

Geometric and cell contact constraints have been suggested to drive invariant development in ascidians with links to asymmetric cell division and cell fate specification^40^. Seam cells are highly dynamic and reconnect to each other after every round of cell division. This cell-to-cell contact has been proposed to be an important cue for cell elongation to stop, thereby allowing reiterative asymmetric seam cell divisions to occur^26^. The importance of cell-to-cell contact in epidermal patterning is exemplified by early studies on neuroblast production from the V5 lineage, which has been shown to require contact of V5 with its neighbouring lineages at the L2 stage^26^. However, outside the V5 lineage, it is not known whether the fate of seam cells and their patterns of divisions would be affected if cell contacts are perturbed. We speculate that the seam cell patterning defects in *eff-1* mutants may be driven by the disrupted physical cell-to-cell communication in the form of the observed gaps in seam cell continuity, which could lead to cell fate changes in neighbouring seam cell lineages. This is consistent with our lineaging analysis, which suggested that 43% of early seam cell losses were linked to seam cell duplications in adjacent lineages at a later development stage. This may also explain the synthetic interaction observed when we combined the *eff-1* mutation with knockdown of *nhr-25,* another background in which loss of seam cell continuity has also been reported^30^. Systematic cell ablations need to be pursued in the future to explore the influence of one cell on the development of its neighbours. It will be also exciting to discover the exact signals that are transmitted between seam cells through their cell-to-cell contact and understand their influence on epidermal tissue homeostasis.

The discovery of increased seam cell number variance in the *eff-1* background is reminiscent of our previous findings on *lin-22,* a Hes-related basic helix-loop-helix (bHLH) transcription factor, which acts in a cell autonomous manner in the seam to mediate robust wild-type seam cell patterning^9,41^. In both mutants, there is a breakdown of seam cell number robustness as evidenced by the observation that phenotypic variance increases. However, this does not mean that these genes have evolved as specific buffers of seam cell patterning, as in both cases they play key epidermal roles in suppressing ectopic neurogenesis or mediating cell fusion. In *eff-1* and *lin-22* null mutants, seam cell gains and losses can occur within the same lineages in the same individual. The developmental basis of seam cell gains is shared between the two mutants, as in both cases it involves symmetrisation of normally asymmetric cell divisions towards the seam cell fate. However, seam cell losses are distinct because *lin-22* mutants make ectopic neurons at the expense of seam cells at the early L2 stage. Taken together, these results indicate that core components of the stem cell maintenance and differentiation network can influence developmental variance by acting both in a cell autonomous or non-cell autonomous manner.

The exact relationship between cell fusion and hypodermal cell differentiation had been so far poorly resolved. It is conceivable that fusion of daughter cells to the syncytial hypodermis allows the reception of differentiation signals upon the breakdown of the cell membrane. A previous study suggested that cells that are unable to fuse remain in developmental limbo displaying lack of commitment to any specific epidermal cell fate^21^. Using a combination of molecular markers, we resolve here that correct fusion is largely not required for cell differentiation in the epidermis, as normal hypodermal marker expression was found to occur in anterior daughters in *eff-1* mutants, while seam cell markers were absent. These results indicate that the differentiation of anterior seam cell daughters into hypodermis occurs normally in *eff-1* mutants, likely as part of an intrinsic differentiation programme. Therefore, cell fusion does not appear to be a signal for cell cycle exit, which is congruent with the observation that *eff-1* mutant cells that fail to fuse do not over proliferate during vulval development^42^. Our lineage analysis is also consistent with the notion that lack of cell fusion does not have detrimental consequences to cell fate allocation as the majority of cell divisions occur normally in *eff-1* mutants, which is remarkable since these mutants are profoundly perturbed in morphology. Nevertheless, lack of cell fusion led occasionally to anterior daughter cells acquiring the seam cell fate, which contributed to an increase in seam cell number. This defect was most frequent at the L4 stage, which may reflect some time-dependent sensitivity of the seam cell lineage towards symmetrisation of divisions towards the seam cell fate, as previously observed in other mutant backgrounds and different temperature environments^9,10^. Although it is not clear what underlies the stochasticity of developmental patterning errors observed, we hypothesise that the accumulation of structural defects breaking the integrity of the epidermis due to breaks in the seam line and bifurcations may be significant contributors.

## Materials and methods

### *C. elegans* culture and maintenance

*C. elegans* was maintained according to standard procedures on NGM plates seeded with OP50 bacteria^43^. All experiments were carried out at 20 °C unless stated otherwise. RNAi plates contained 50 µg/ml ampicillin,12.5 µg/ml tetracycline and 1 mM filter-sterilised isopropyl β-D-1-thiogalactopyranoside (IPTG) for dsRNA induction. Homozygous lines were created in all cases except for panels 6A, B where F1s were visualised from a cross between MBA226 and AW298 (wild-type) or MBA251 and MBA202 (*eff-1* mutant). Seam cells were visualised using the *scm::GFP* marker (*wIs51*)^19^. To be able to visualise the seam cell membrane, membrane-targeted GFP::CAAX or GFP::PH were driven in the seam cells using the last intron of *arf-5* together with a minimal *pes-10* promoter^9^ or the *wrt-2* promoter^27^ respectively. Seam cell nuclei were visualised with *wIs51* or GFP::H2B under a *wrt-2* promoter^27^. All strains used in this study are listed in Supplemental Table S1.

### Chemical mutagenesis screen and mutation mapping

Mutagenesis and screening have been previously described^9^. Briefly, synchronised JR667 L4 larvae were incubated in 50 mM EMS in M9 buffer for 4 h. F2s were screened for aberrant seam cell number (SCN ≤ 14 or ≥ 18) under an Axio Zoom.V16 (Zeiss) fluorescent dissecting microscope and the mutant phenotype was confirmed in the F3 generation. Lines that showed two-sided errors and increase in phenotypic variability were chosen for further analysis. Mutant strains like MBA21 were outcrossed 4 times before phenotypic characterisation.

To map the *icb4* mutation, MBA21 was crossed to males of the polymorphic Hawaiian strain (CB4856). The F1 hermaphrodites from a successful cross were allowed to self. F2 animals were screened for aberrant seam cell phenotype and placed individually on NGM plates. F3 animals were scored to validate the variable seam cell number phenotype. Once the *E. coli* food was consumed, animals were washed off the plate in M9 buffer and were stored as a pellet at − 20 °C until DNA extraction. The lines showing the most consistent variable seam cell number phenotype were pooled together and their DNA was extracted using a Gentra Puregene Kit (Qiagen). Whole genome sequencing was outsourced at Eurofins and was performed using an Illumina Hiseq platform to reach 20 × genome coverage.

### Phenotypic analysis and microscopy

Fluorescence microscopy was performed to quantify seam cell number at the 1-day old adult stage. Animals were mounted on fresh 2% agarose pads and were immobilised using 100 µM sodium azide. Seam cell were scored on one lateral side using a 40 × objective on an AxioScope A1 (Zeiss) compound microscope using an LED source and a GFP filter. One-way or two-way ANOVA was conducted to test for differences in the mean seam cell number. When there was a significant effect of strain/treatment on seam cell number, post hoc Tukey HSD tests were conducted. Levene’s median test was used to test for differences in variance in seam cell number between strains.

For scanning electron microscopy (SEM) microscopy, 1-day old adults were collected and washed twice with M9 buffer, before fixation in 4% glutaraldehyde in M9 for 3 h at room temperature. Fixed animals were washed twice with M9 buffer and then were gradually dehydrated by incubation for 30 min in an ethanol solution starting from 15% and moving towards 100%. Animals were washed in 100% ethanol another 4 times over a period of two days. Samples were then dried using a critical point dryer (K850, ProSciTech) and coated with gold/palladium using the SC7620 Mini Sputter Coater (Quorum technologies). Imaging was performed on a JEOL JSM-6390 scanning electron microscope using 5–25 kV of acceleration voltage.

Long-term time-lapse microscopy of *eff-1* was performed as previously described^28^. In total, 11 *eff-1(icb4)* animals were imaged in microchambers throughout their development. 2 animals were excluded from the analysis because of poor imaging quality so our data correspond to 18 seam cell lineages (i.e. two lateral sides of the remaining 9 animals).

Single molecule FISH was performed in synchronised fixed animals as previously described^9,10^. Imaging was performed on a Nikon Ti Eclipse epifluorescence microscope using the 100 × objective and an Andor iKon M934 CCD camera system. The Cy5 labelled oligos (Biomers) included in each probe are shown in Supplemental Table S2.

To perform seam cell shape and cell alignment analysis, images were acquired using an oil immersion 40 × objective with a CoolSNAP HQ Monochrome camera (Photometrics,USA), Animals were synchronised and imaged 17 h (late L1 or L2 symmetric division) and 24 h (asymmetric division) after bleaching. Images were straightened using a semi-automatic ImageJ pipeline^44^, and cells were segmented to extract cell shape parameters of major cell axis, minor axis, perimeter and area. To quantify the degree of misalignment between any anterior and posterior cell, a horizontal line (parallel to body axis in a straightened image) was drawn to intersect the centroid of the anterior cells and another line to connect the centroids of both cells, between which the magnitude of the corresponding angle was measured. One-way analysis of variance (ANOVA) was performed on magnitude of angles between the strains. The genotype was considered the explanatory variable and the angle as the dependent variable. Principle component analysis (PCA) was carried out using the prcomp function in the R software environment^45^. PCA was performed on four cell shape parameters (minor, major, perimeter and area) of individual seam cells at the end of L1 asymmetric division.

To measure the angle of cell division, we used a strain expressing GFP::PH and GFP::H2B under the *wrt-2* promoter^27^. The angle between the horizontal line parallel to the longitudinal axis of the cell drawn to intersect the segregating DNA of the anterior cell and the line connecting the segregating DNA of dividing cells was measured at the L2 symmetric and asymmetric division stage.

## Supplementary Information

Supplementary Information
