# Supplementary material for: A role for the fusogen eff-1 in epidermal stem cell number robustness in Caenorhabditis elegans
Source: Sci Rep. 2021 May 7;11:9787. doi: 10.1038/s41598-021-88500-4 (PMC8105389; doi:10.1038/s41598-021-88500-4)
Supplement: Supplementary file 1 — Supplementary Information [file 41598_2021_88500_MOESM1_ESM.pdf]

Supplemental Information for:

A role for the fusogen *eff-1* in epidermal stem cell number robustness in *Caenorhabditis elegans*

Sneha L. Koneru[1], Fu Xiang Quah[1], Ritobrata Ghose[1,3], Mark Hintze[1], Nicola Gritti[2], Jeroen Sebastiaan van Zon[2] and Michalis Barkoulas[1,★]

[1] Department of Life Sciences, Imperial College, London SW7 2AZ, United Kingdom.

[2] AMOLF, Science Park 104, 1098 XG Amsterdam, the Netherlands

[3] Present address: Centre for Genomic Regulation (CRG), The Barcelona Institute of Science and Technology, Barcelona, 08003, Spain

★Correspondence: m.barkoulas@imperial.ac.uk

Supplemental Figures 1-5

Supplemental Tables 1-2

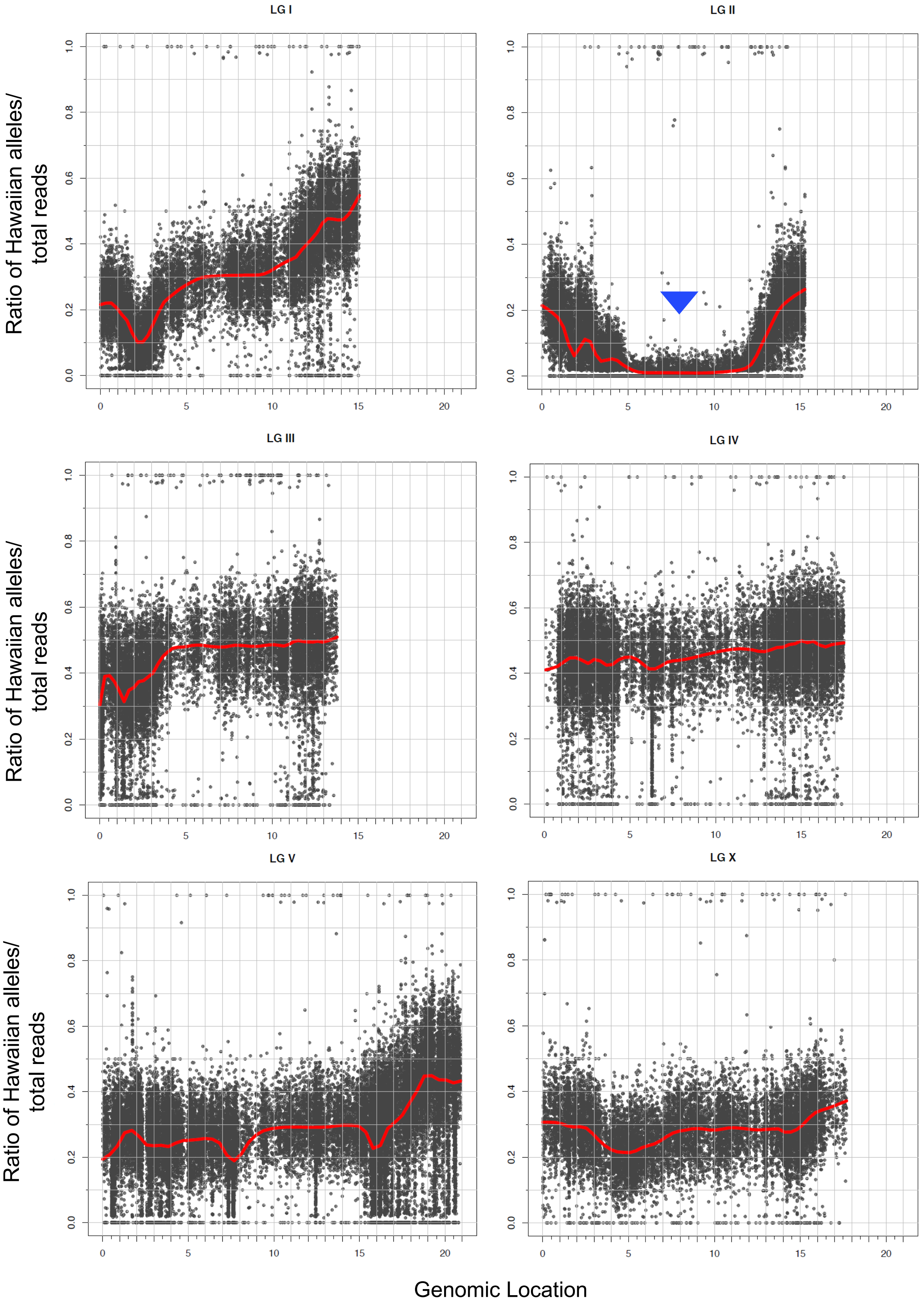
LG I
LG II
LG III
LG IV
LG V
LG X
Ratio of Hawaiian alleles/
total reads
Genomic Location

Figure S1

Figure S1: The *icb4* mutation in strain MBA21 maps to the middle of chromosome II. Ratio of CB4856 alleles to total number of sequencing reads along the six chromosomes. Red curves represent locally weighted scatterplot smoothing (LOESS) regression lines from the allele frequencies at known SNP positions along the chromosomes with a span parameter of 0.1. Blue arrow points to region with enrichment of N2 reads on chromosome II between 6 Mb – 10 Mb, where the causative mutation resides.

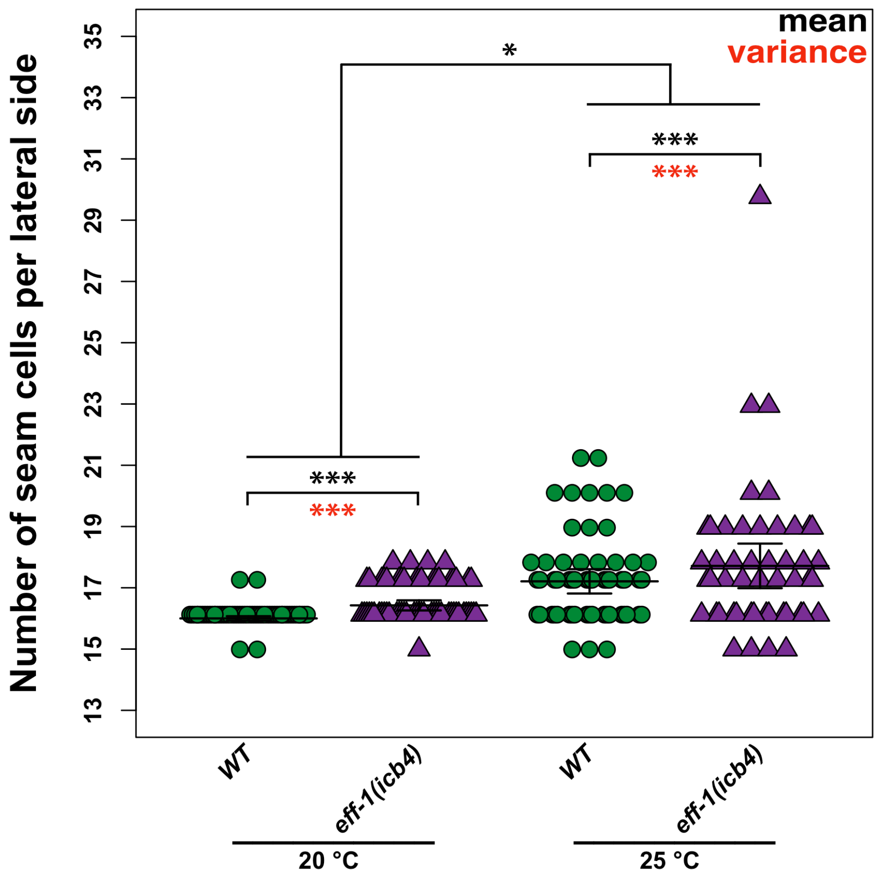

mean
variance
Number of seam cells per lateral side
35
33
31
29
27
25
23
21
19
17
15
13
*
***
***
***
***
WT
eff-1(icb4)
WT
eff-1(icb4)
20 °C
25 °C

Figure S2

Figure S2: Seam cell counts of *eff-1(icb4)* scorings at 25 °C

(a) Increase in seam cell variability in *eff-1(icb4)* mutants compared to wild-type animals is observed at both 20 °C and 25 °C. Culture at 25 °C increases mean seam cell number in both WT and *icb4*. A two-way ANOVA showed that the effect of strain and temperature on SCN is significant (Strain - $F(1, 219) = 39.28$, $p = 1.93 \times 10^{-9}$, Temperature - $F(1, 219) = 5.64$, $p = 1.84 \times 10^{-2}$). $49 \leq n \leq 61$. Error bars indicate 95% confidence intervals. Black stars show statistically significant changes in the average seam cell number by post hoc Tukey's HSD test, and red stars depict changes in variance with a Levene's median test (*** corresponds to p value $< 1 \times 10^{-4}$, ** $p < 1 \times 10^{-3}$, * $p < 0.05$).

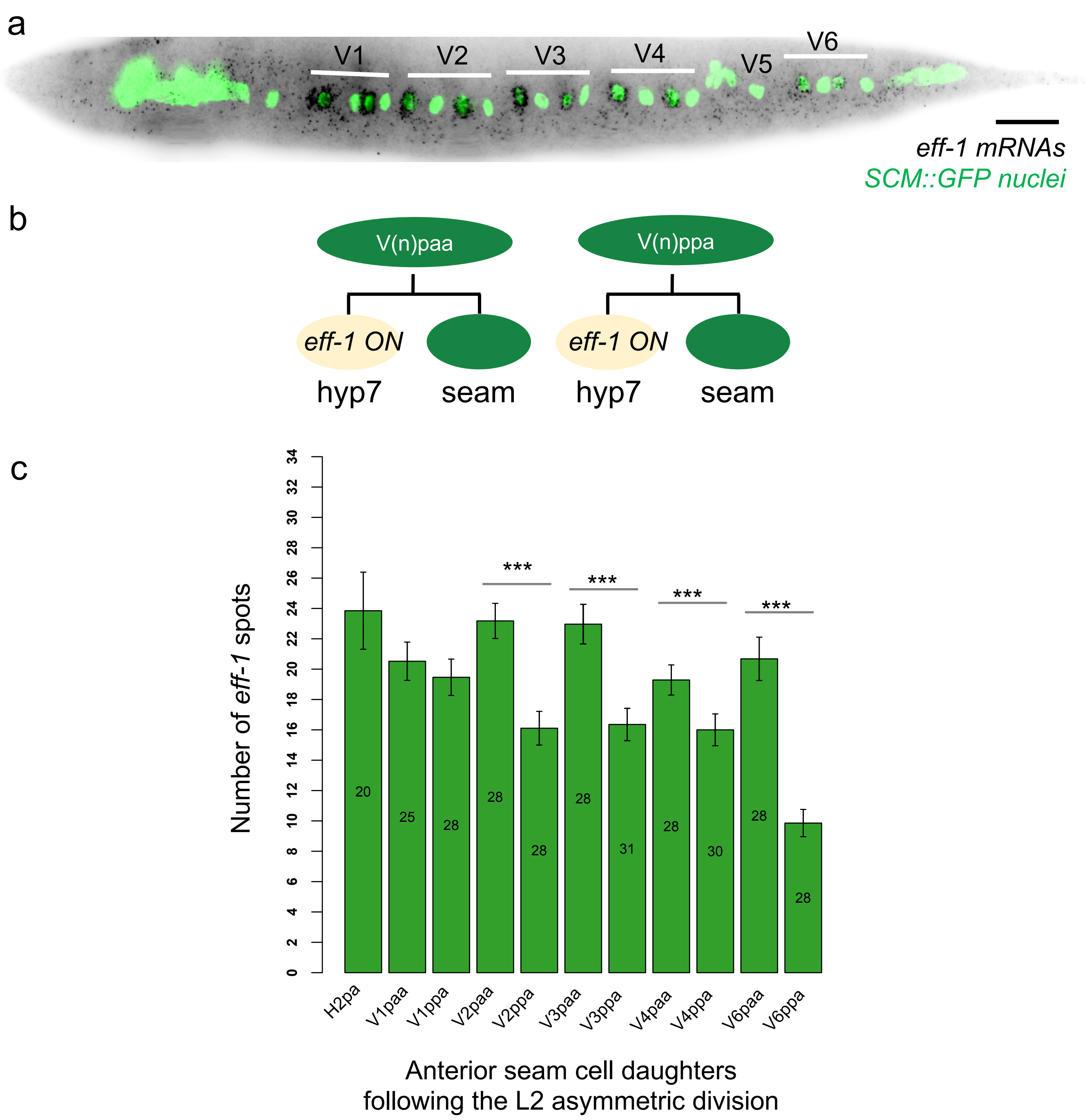
a
V1
V2
V3
V4
V5
V6
eff-1 mRNAs
SCM::GFP nuclei
b
V(n)paa
V(n)ppa
eff-1 ON
eff-1 ON
hyp7
seam
hyp7
seam
c
Number of eff-1 spots
0
2
4
6
8
10
12
14
16
18
20
22
24
26
28
30
32
34
***
***
***
***
20
25
28
28
28
28
31
28
30
28
28
H2pa
V1paa
V1ppa
V2paa
V2ppa
V3paa
V3ppa
V4paa
V4ppa
V6paa
V6ppa
Anterior seam cell daughters
following the L2 asymmetric division

Figure S3

Figure S3: *eff-1* is expressed in anterior seam cell daughters.

(a) Representative smFISH image showing *eff-1* expression in the wild-type after the L2 asymmetric seam cell division. Seam cells are labelled in green using the *scm::GFP* marker. Black spots correspond to *eff-1* mRNAs. Scale bar is 20 μm. (b) Cartoon showing *eff-1* expression in V cell daughter pairs at the stage in which mRNAs were measured by smFISH. (c) Quantification of *eff-1* mRNAs in anterior seam cells following the L2 asymmetric division. Note that anterior cells at the anterior branch of the V2, V3, V4 and V6 lineage appear to express more *eff-1.* Posterior daughter cells, which do not express *eff-1*, are not shown. Black stars indicate statistically significant differences in *eff-1* expression with pairwise t-test (*** corresponds to p value < 1×10^{-4}). Error bars indicate 95% confidence intervals. Number of cells analysed is shown inside the bars ($20 \le n \le 31$).

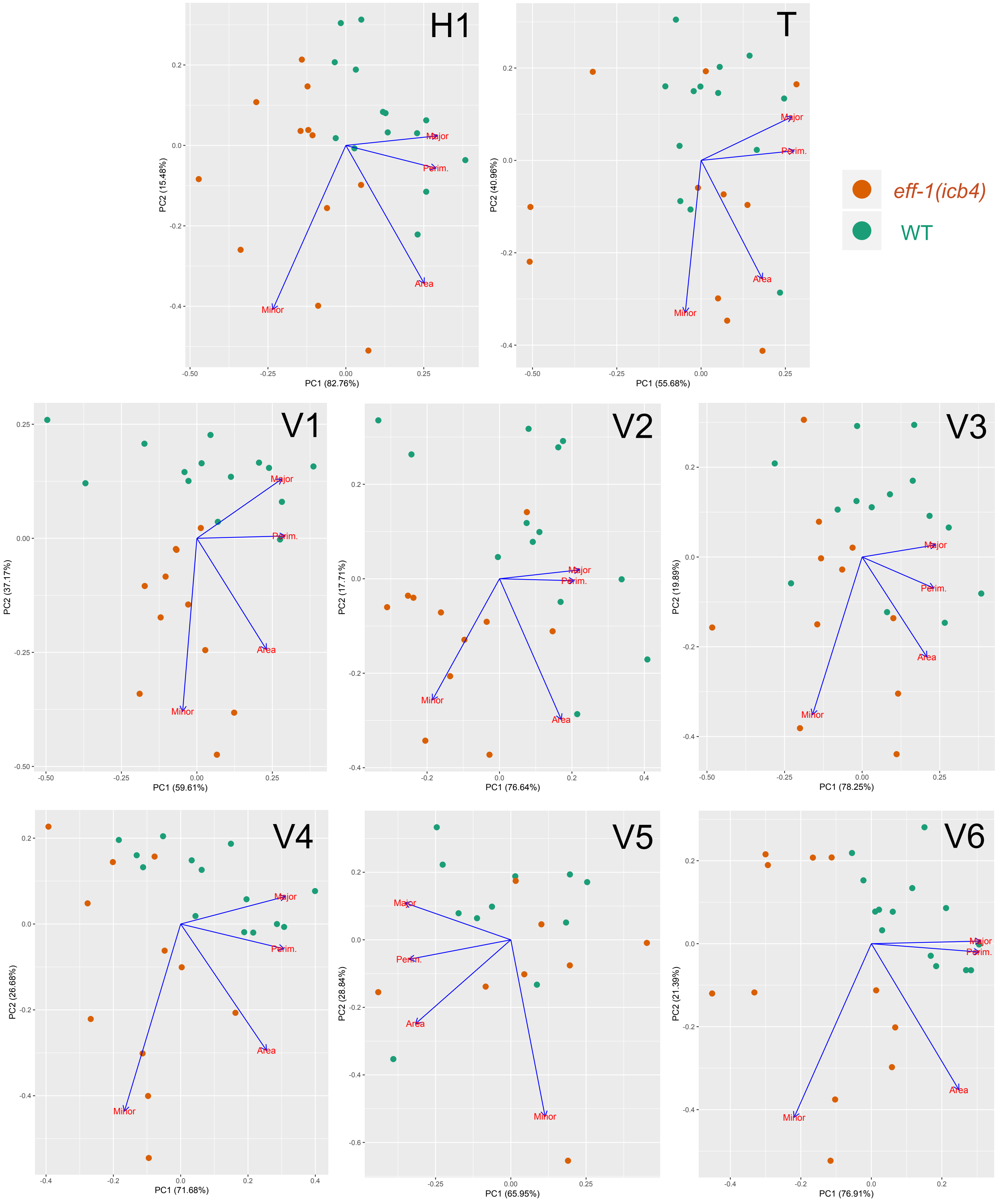
H1
T
V1
V2
V3
V4
V5
V6
Major
Perim.
Area
Minor
PC1 (82.76%)
PC2 (15.48%)
PC1 (55.68%)
PC2 (40.96%)
PC1 (59.61%)
PC2 (37.17%)
PC1 (76.64%)
PC2 (17.71%)
PC1 (78.25%)
PC2 (19.89%)
PC1 (71.68%)
PC2 (26.68%)
PC1 (65.95%)
PC2 (28.84%)
PC1 (76.91%)
PC2 (21.39%)
eff-1(icb4)
WT

Figure S4

Figure S4: Quantitative comparison of seam cell shape between wild-type and *eff-1(icb4)* mutants after the L1 stage division.

Principle component analysis is shown for H1, V1-V6 and T cells independently. Green and orange dots correspond to seam cells in wild-type and *eff-1(icb4)* respectively. Individual cells are plotted with respect to first and second principal components, which account for more than > 90% of the total variance. Arrows represent variables used in the analysis and point in the direction of increasing values of that variable.

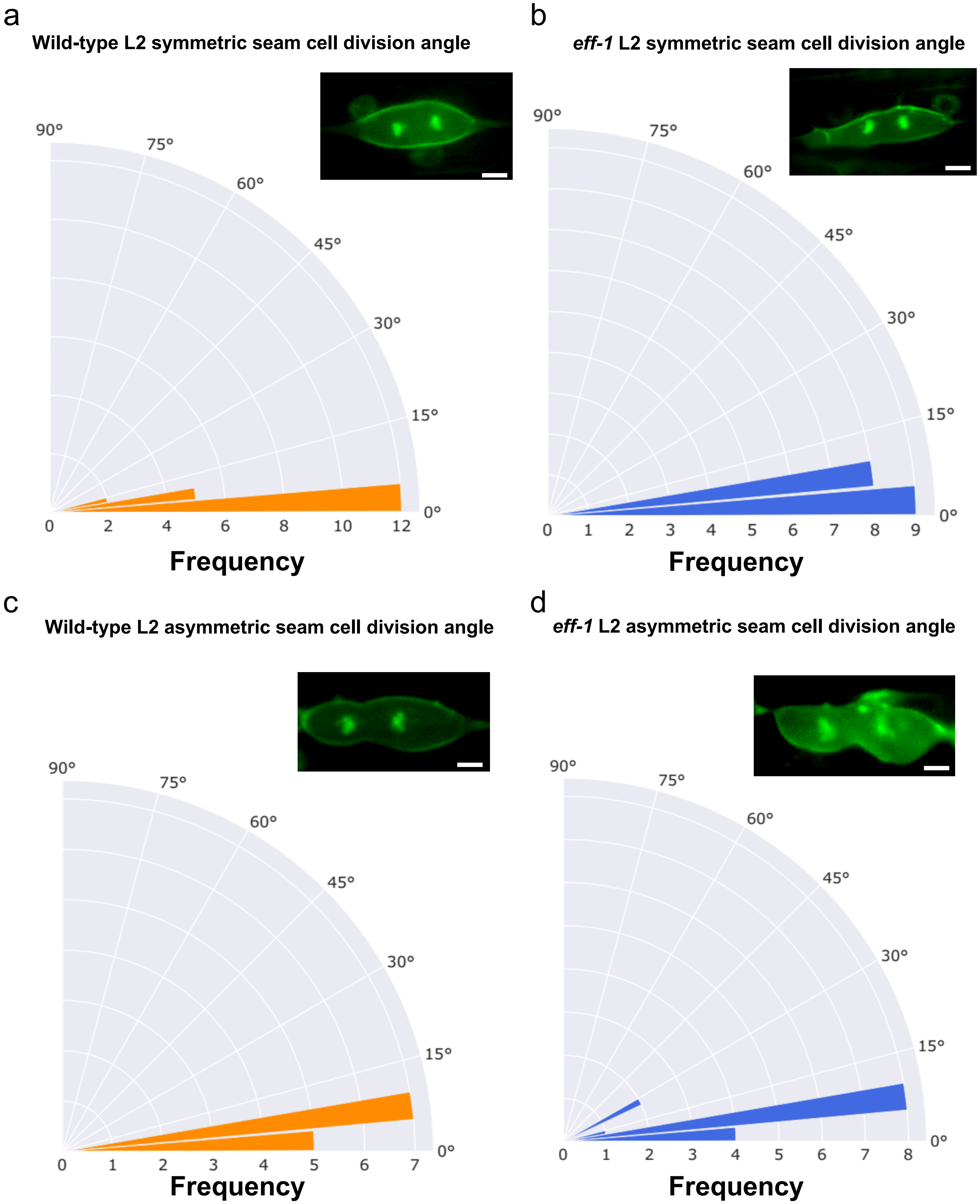
a
Wild-type L2 symmetric seam cell division angle
90°
75°
60°
45°
30°
15°
0°
0
2
4
6
8
10
12
Frequency
b
eff-1 L2 symmetric seam cell division angle
90°
75°
60°
45°
30°
15°
0°
0
1
2
3
4
5
6
7
8
9
Frequency
c
Wild-type L2 asymmetric seam cell division angle
90°
75°
60°
45°
30°
15°
0°
0
1
2
3
4
5
6
7
Frequency
d
eff-1 L2 asymmetric seam cell division angle
90°
75°
60°
45°
30°
15°
0°
0
1
2
3
4
5
6
7
8
Frequency

Figure S5

Figure S5: Angle of V lineage seam cell division at symmetric and asymmetric L2 cell division. (a-d) The angle between condensed chromatin at telophase and the longitudinal axis of the cell in symmetric (a-b) and asymmetric (c-d) cell division. No significant difference was found between wild type and *eff-1(icb4)* mutants (two-tailed t-test $p>0.15$). Scale bars in a-d represent 2.5 μm. Images show expression of *wrt-2p::GFP::H2B* and *wrt-2p::GFP::PH* used to visualise the seam cell membrane and segregating DNA during anaphase in the two daughter cells.

Supplemental table S1: List of strains used in this study

Strain	Genotype
JR667	*unc-119(e2498::Tc1) III; wIs51[scm::GFP + unc-119(+)] V*
CB4856	Hawaiian wild isolate *C. elegans*
AW298	*wIs78 [scm::gfp + ajm-1::gfp + unc-119+] IV; him-5(e1490) V*
MBA21	*wIs51; eff-1(icb4)* II
MBA79	*wIs51; eff-1(icb4) II* (backcrossed 4x times)
BP328	*eff-1(ok1021) II; hmIs4[des-2::GFP + pRF4(rol-6(su1006))].*
MBA273	*eff-1(ok1021) II; wIs51[SCMp::GFP + unc-119(+)] V*
BP75	*eff-1(hy21)II*
MBA156	*eff-1(hy21) II; wIs51[SCMp::GFP + unc-119(+)] V*
MBA178	*wIs78 [scm::gfp + ajm-1::gfp + unc-119+] IV*
MBA187	*eff-1(icb4) II; wIs78 [scm::gfp + ajm-1::gfp + unc-119+] IV*
MBA237	*icbIs3[arf-3::GFP:CAAX::unc-54] III; wIs51[SCMp::GFP + unc-119(+)] V*
MBA246	*eff-1(icb4) II; icbIs3[arf-3::GFP:CAAX::unc-54] III; wIs51[SCMp::GFP + unc-119(+)] V*
MBA247	*icbIs3[arf-3::GFP:CAAX::unc-54] III; icbSi2[dpy-7::mCherry::H2B::unc-54 3'UTR+cb-unc-119] IV; wIs51[SCMp::GFP + unc-119(+)] V*
MBA252	*icbIs2[arf-3::GFP:CAAX::unc-54] I; eff-1(icb4) II; icbSi2[dpy-7::mCherry::H2B::unc-54 3'UTR+cb-unc-119] IV; wIs51[SCMp::GFP + unc-119(+)] V*
MBA226	*unc-119(ed3) III; icbSi2[dpy-7::mCherry::H2B::unc-54 3'UTR+cb-unc-119]*
MBA202	*eff-1(icb4) II; wIs78 [scm::gfp + ajm-1::gfp + unc-119+] IV; him-5(e1490) V*
MBA251	*eff-1(icb4) II; icbSi2[dpy-7::mCherry::H2B::unc-54 3'UTR+cb-unc-119] IV; wIs51[SCMp::GFP + unc-119(+)] V*
MBA459	*icbIs2[arf-3::GFP:CAAX::unc-54] I; eff-1(icb4)II; egl-18(ga97) IV; wIs51[SCMp::GFP + unc-119(+)] V*
MBA290	*egl-18(ga97) IV; wIs51[SCMp::GFP + unc-119(+)] V*
SV10009	*heIs63[wrt-2::GFP::PH; wrt-2::GFP::H2B; plin-48::mCherry]*
MBA462	*eff-1(icb4) II; heIs63[wrt-2::GFP::PH; wrt-2::GFP::H2B; plin-48::mCherry]*

Supplemental table S2: List of smFISH probes used in this study

**Single molecule fluorescent *in situ* hybridisation probes used in this study**			
eff-1	***egl-18***	***elt-3***	***nhr-73***
aactggggagaccactcaaa	cgtcattatgctgatcgaca	cggaaagttgagagtccttc	tatttcattactcggtctcc
aatccgtaggttgtaactgc	agcacttcgtggtgttgttg	acagtaatatgcagctggtt	ctgtgacaaacttggcagga
atcgaatttctcctcgagtg	ctacacggctcatctgacgg	actcgataatcgttcatcgt	aaagtacaggcgggttcgtt
tgtcttggaacagtgtggtg	cttctgtaactgtttgcaac	tagtgtgttggccactttttt	aagctgcgcaggaggtgatg
gagatgtttgagcacggaca	tgctgattgtctttgcaaca	ggtcaagttgaacatatgga	cggatcttcggaagaatgca
gaattgcatttgcattcctc	ttgtccattcgctccataac	ttagtgaagtccgcataggt	cagtgcacatgtagcgaatc
atctgaagcagactgcagtg	ctcgtcgagccgatactgaa	ctgatggttcagtacctgtg	gaaatagtgcagctgttagt
tcattgatctcttgggatgc	gctcattgttctctttgagc	agttcatctggagcatttct	tgcatgctctgcagaagaac
atgtctgatttccagcattt	gatgagaccgatgagctttt	cttgtggataatggtgttgg	ggctcgaatacaactggtgt
gttcaagcttttccaatcga	cggtgatggtgaggcttttc	gtgacttgtgctgtattcat	cattacggctcatgaccatc
aagtgtaccgctgagttatc	tctcgacaagcttcggagag	gaacggcatttgatgtgtct	gcttttctagcagtagctaa
tggcatgaacttcagggatt	cctgatactggagcgactac	tgcgaagtttgaaaggttca	ccgtggtattatgttgttcg
tggcatcacactcacagata	aagtctggaagtggactcgc	tgtcacaggctggttggaat	gtctacttcctcaagtgacg
agattctgcggtacatgttg	ggatcaaacatgaatccgtt	gctgttgagaagtggaaggg	tgttcaggatcttgctgaac
agactctggacaagcggtaa	gcatcattccatttggattt	tgttgtgtatggatgcgttg	acttgagcaaatccccgttg
gcggtagcatgaagatgttt	ctcacggattgttgattctc	gtggaggtgtgaagtttgac	ctctccacttctttcacata
tggtgtctgatttgggaaga	cacggatttcgattgtggtg	ttcttttttcatcggcttc	atatctctgctgagccatta
tcgaacgtcacagcaaagct	gatccattggatcttcaatt	tgatgacattgtacagctgc	tggagagcgttcttttgctc
gtttgactgcgaggaatgtc	gactcttcctgtttcacatc	gtctcacgtgttttgcaatt	aagatcgttcactgacttca
atgttgcatacgttgtaggt	agtgcatccaaaaggttctg	aagtagagattgcaggcgtt	cttggtaggcggtgacaatc
tttatctttttccacccaat	gttgctgctaaactgtgctg	tccatcttttctaagagaca	tgaagcacgattccagggat
tgtgttccaccatctaattg	tggtggaggtctggaagaac	gacgtcggtttctcttcata	acagtatatctactccaggg
cgacgtttttggtcgagatg	gatgatgatgatggtggaga	tgcagagtttggagactcat	cacaacctcaacgtcttcat
ggcagttacagccaatgaaa	ttcgatgaccgcttgtactt	catgtctctgatgggtattc	aatccaggtgttcgagaact
cagttgatgagatgctcgtc	ttgaactgtcttggctttgg		gaagtaggtgcaaggagtga
ctccattacttgttcttgag	ctcgtctcggttttctcaaa		gttacagctaggatccagtg
tcaatggttgcattctcagt	acggattgcagacaagcttc		ggatgaactcagagattctt
accatccaagacggtcaaag	gtgcaatcgatagtagagcc		cccagagttgacttgagtag
atgaccagaatcgtccattc	atttctattggtcggcgaac		ttcaggttaagctgggcaag
tccattttcacaactccatt	ttgttggatgtggtttttgc		gaaggctgcgtattcaatga
gcaatttttcactttagcct	actctttttccttgttcttt		gcttccagatgcaaaatgac
tcgagcagattgaatccacg	tggttgaagatctgtgttgc		atgcttgttttgtgatacac
gtgttacaacagcttgtctg	atcgtcggcatttgtgtgag		ctgagccacaatcttcattg
tgaagattagttccttcggc	ttgaatgtgttgatggctcc		gcacttgtaactccttcata
gacaaggttttgactttcca	ctgcgtgaattgcgagattt		gttcttcctataatacttcc
gatggatccactgaagtcac	tgctattcatgagctcttga		ccatttccaattcactcatt
agcctcatatactgtcaagt			tgtgatatccccgattcttg
ctgatccatcaatttttcca			tcgatacagtgatgatttgc
tccaaatccagttgacatct			cgagccatgtcattgtagag
atggctggcagtggaataat			aagcccagtttgatgataca
tgaatctgctcggagacaga			
tttccaaagggctctcgaaa			
ccattttccctcaacaagat			
attacaagttgggcaggttc			
gatccaatgagctggattca			
caccaatcgaactgattcct			
atccgtagcaatcataacgc			
gacaaatttgtcccaacgga			
